# The Role of PLATZ6 in Raffinose Family Oligosaccharides Loading of Leaves via PLATZ Family Characterization in Cucumber

**DOI:** 10.3390/plants13192825

**Published:** 2024-10-09

**Authors:** Peiqi Wang, Haofeng Teng, Dan Qiao, Fei Liang, Kaikai Zhu, Minmin Miao, Bing Hua

**Affiliations:** 1 College of Horticulture and Landscape Architecture, Yangzhou University, Yangzhou 225009, China; m15252577369@163.com (P.W.); thf2193228179@163.com (H.T.); qiaod7201@163.com (D.Q.); 15805235039@163.com (F.L.); mmmiao@yzu.edu.cn (M.M.); 2 Co-Innovation Center for Sustainable Forestry in Southern China, Nanjing Forestry University, Nanjing 210037, China; kkzhu@njfu.edu.cn

**Keywords:** cucumber, PLATZ, STS, GolS1, RFOs

## Abstract

The *plant AT protein and zinc-binding protein* (*PLATZ*) genes, a novel cluster of plant-specific zinc-finger-dependent DNA-binding proteins, play a crucial role in regulating stress response and plant development. However, there has been little study focus on the role of the cucumber PLATZ family in assimilating loading in leaves. (1) In this study, a total of 12 *PLATZ* genes were identified from the cucumber genome. The cucumber *PLATZ* genes were clustered into five groups, and unevenly distributed on five chromosomes. A single pair of cucumber *PLATZ* genes underwent segmental duplication. (2) The results of genome-wide expression analysis suggested that the cucumber *PLATZ* genes were widely expressed in a wide range of cucumber tissues, with three *PLATZ* (*PLATZ2*, *PLATZ6*, and *PLATZ12*) genes exhibiting high expression in the vascular tissues of cucumber leaves. *PLATZ2*, *PLATZ6*, and *PLATZ12* proteins were primarily located in cytomembrane and nucleus. (3) In VIGS-*PLATZ6* plants, the expression of *Galactinol synthase 1* (*GolS1*) and *STACHYOSE SYNTHASE* (*STS*), two genes involved in the synthesis of raffinose family oligosaccharides (RFOs) were observed to be decreased in cucumber leaves. In conclusion, the comprehensive analysis of the cucumber PLATZ family and the preliminary functional verification of PLATZ6 lay the foundation for the molecular and physiological functions of cucumber *PLATZ* genes.

## 1. Introduction

In plants, photosynthesis fixed carbon and the assimilation products are exported to sink organs for their growth and development. Sucrose (Suc) is the predominant assimilation product transported in the vascular bundle of leaves in most plants [1,2]. In some plant species, including cucumber (*Cucumis sativus* L.), a globally significant vegetable being of considerable great nutritional and economic value, raffinose family oligosaccharides (RFOs) are the major assimilation products transported between the “source” and “sink” organs [3,4].

In plants, three loading strategies have been proposed: metabolically active loading from the apoplast, passive loading via the symplast, and symplastic transfer followed by polymer trapping of RFOs [5]. In apoplastic loading, sieve element-companion cell complexes are symplastically isolated from the surrounding cells [6]. In the apoplastic loading pathway, sucrose is synthesized in the mesophyll cells and subsequently moved into phloem parenchyma cells via symplastic transport [7]. A number of sucrose transporters (SUTs) have been identified. SWEETs are the primary sucrose transporters [8,9]. In symplastic loading, sieve element-companion cell complexes are connected to mesophyll cells by plasmodesmata, and photoassimilated products diffuse into the phloem without crossing the plasma membranes [9,10,11]. In polymer-trapping loading plants, sucrose is synthesized in mesophyll cells and diffuses into intermediary cells (ICs) through plasmodesma, and sucrose is converted to RFOs and then transferred to sieve elements for long-distance transport through the phloem [12]. In the RFO-synthesizing pathway, galactinol synthase (GolS), raffinose synthase (RS), and stachyose synthase (STS) are the key enzymes [3,13,14,15,16]. GolS catalyzes the initial enzyme in RFO biosynthesis, catalyzing the conversion of UDP-galactose and myo-inositol into galactinol, which serves as the galactosyl donor for RFO synthesis; RS transfers a galactosyl group of galactinol to sucrose and catalyzes raffinose formation; STS catalyzes raffinose and galactinol to synthesize stachyose [17,18,19,20,21,22].

In plants, cucumber and *Verbascum phoeniceum* (*V. phoeniceum*) are typical polymer-trapping loading specie. In *V. phoeniceum*, the suppression of the expression of two galactinol synthase genes (GAS), *VpGAS1* and *VpGAS2,* which are localized in ICs, repressed phloem loading [13]. In cucumber, the suppression of the cucumber *STS* gene reduced low-temperature stress tolerance and inhibited phloem loading [16]. The cucumber *STS* gene is regulated by its cis-antisense RNA, *asCsSTS*, which is involved in balancing source–sink carbon partitioning [4]. Although the synthetase involved in RFO synthesis has been well identified, the regulatory gene of RFO synthetase has rarely been reported on.

So far, a set of genes has been identified to regulate the synthesis and storage of assimilation products. Among them, plant AT protein and zinc-binding protein (PLATZ) transcription factors are involved in plant development, environmental signaling, stress response, and starch synthesis. The PLATZ protein family was initially identified in the pea (*Pisum sativum*) plant and represents a novel class of zinc finger proteins that are unique to plants and contain conserved PLATZ domains. PLATZ proteins have been demonstrated to bind A/T-rich sequences and inhibit gene transcription [23]. To date, the PLATZ family has been identified in several species, including 15 members in *Oryza sativa*, 13 members in Arabidopsis, 17 members in *Zea may*, 24 members in tomato, and 11 members in watermelon [24,25,26,27]. A set of studies have shown that PLATZ proteins are involved in plant growth, development, and abiotic stress. In maize (*Zea mays*), *Floury3* (*FL3*) exhibited a high expression in starch endosperm cells and regulated the RNAPIII transcriptional machinery by interacting with transcription factor class C 1 (TFC1) and RNA polymerase III subunit 53 (RPC53) [28]. *ZmPLATZ2* was up-regulated by glucose and bound to the promoters of the starch synthetase, which acted as the active regulator of starch synthesis of the Glu signal pathway [26]. *ORE15* (*ORESARA15*), a PLATZ gene of Arabidopsis, participated in regulating leaf senescence and size via *GRF1* and *GRF4*, two key genes of the GROWTH-REGULATING FACTOR (GRF)/GRF-INTERACTING FACTOR (GIF) pathway [29]. In rice, the PLATZ gene *GL6* regulated grain development and cell proliferation [30].

The pivotal role of PLATZ in plant growth and development has been elucidated; however, the role of the cucumber PLATZ gene family in RFO synthesis and loading in leaves remains unclear. In this study, a total of twelve cucumber PLATZ genes were identified in the cucumber genome. To gain further insights into the PLATZ family in cucumber, gene composition, chromosomal location, synteny, and phylogenetic analyses were conducted. The PLATZ genes that are specifically expressed in the vascular tissues of leaves were identified. The suppression of the expression of *PLATZ6* by VIGS resulted in the downregulation of *GolS1* and *STS* expression. This suggests that PLATZ6 may act as a regulator of assimilation product loading in cucumber leaves.

## 2. Results

### 2.1. Identification of PLATZ Family Genes in Cucumber

The identification of PLATZ genes in cucumber was carried out by utilizing the BLASTP tool (http://cucurbitgenomics.org/blast (accessed on 1 September 2023)) with the PLATZ protein sequences of Arabidopsis against the cucumber genome database (Cucumber (Chinese Long) v3 Genome). Additionally, a hidden Markov model (HMM) search with the PLATZ domain (PF04640) was conducted, and 12 *PLATZ* genes were definitively identified in the cucumber genome (Table 1).

To characterize the physicochemical properties of cucumber PLATZ proteins, ExPASy (https://web.expasy.org/ (accessed on 5 August 2023) was used to analyzes the amino acid sequences of cucumber PLATZ proteins. As shown in Table 1, the open reading frames (ORFs) of cucumber *PLATZ* genes ranged from 423 bp for PLATZ9 to 849 bp for PLATZ12. The molecular weights (MWs) of cucumber PLATZ proteins ranged from 16.60 kD (PLATZ9) to 32.32 kD (PLATZ12) (Table 1). The average MW of cucumber PLATZ proteins was 25.48 kD, with a difference value between the maximum and minimum numbers of 16.72 kD (Table 1). Eleven cucumber PLATZ (PLATZ1-11) proteins were identified as acidic proteins (pI less than seven) and PLATZ12 was classified as a basic protein (pI higher than seven) in the cucumber PLATZ family (Table 1). The instability indexes of cucumber PLATZ proteins ranged from 51.31 (PLATZ10) to 84.77 (PLATZ2), indicating that these PLATZ proteins were unstable proteins, as their instability indexes were greater than 40 (Table 1). The aliphatic indexes of cucumber PLATZ proteins ranged from 58.43 (PLATZ9) to 78.09 (PLATZ12) (Table 1). The subcellular localization of cucumber PLATZ proteins was predicted to be in the nucleus using Cell-PLoc (http://www.csbio.sjtu.edu.cn/bioinf/Cell-PLoc-2/ (accessed on 5 August 2023)) (Table 1).

**Figure 1:**
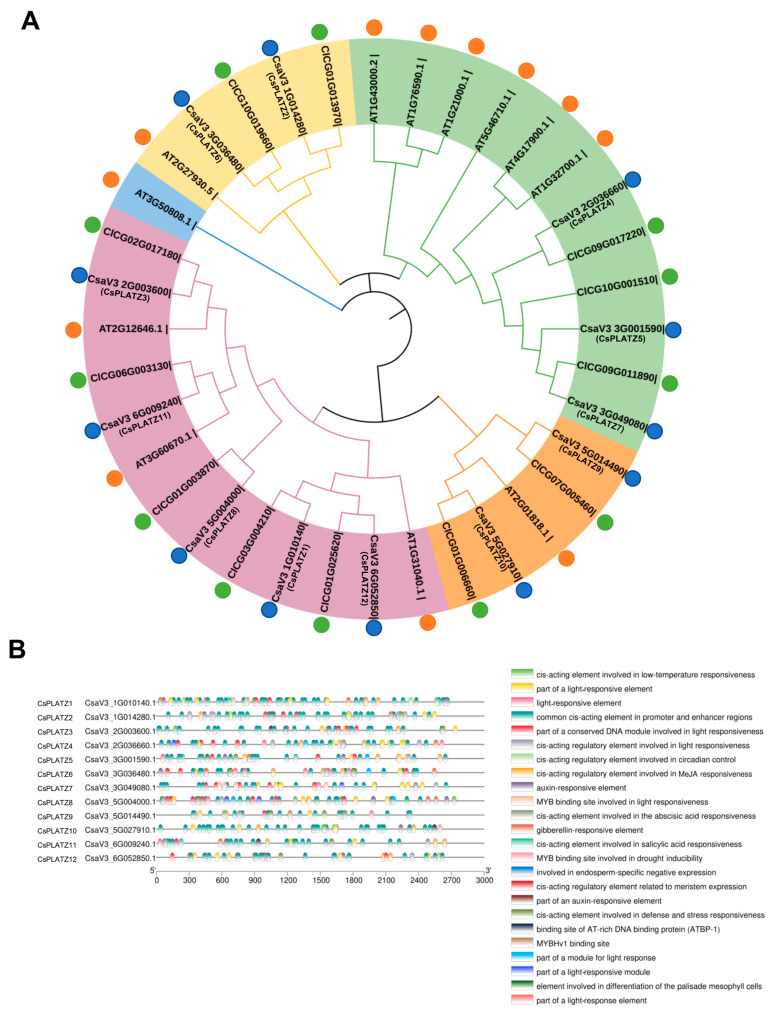
(**A**) Dendrogram of PLATZ family proteins in cucumber, Arabidopsis and watermelon. Twelve PLATZ proteins of cucumber (marked with blue circles), twelve Arabidopsis PLATZ proteins (marked with orange circles), and twelve PLATZ proteins of watermelon marked with green circles were used for the construction of the phylogenetic tree. Trees in different colors represent distinct subgroups. (**B**) CRE (cis-acting regulatory element) analysis in 3000 bp promoter regions of cucumber *PLATZ* genes. The different colored boxes indicate different CRE analyzed with MEME.

The chromosomal distribution of the cucumber PLATZ genes revealed that the cucumber PLATZ genes were located in five chromosomes (chr1, 2, 3, 5, 6). Among the five chromosomes, *PLATZ1* and *PLATZ2* were located on chromosome1; *PLATZ3* and *PLATZ4* were located on chromosome2; *PLATZ5*, *PLATZ6* and *PLATZ7* were located on chromosome3; *PLATZ8*, *PLATZ9* and *PLATZ10* were located on chromosome 5; *PLATZ11* and *PLATZ12* were located on chromosome 6 (Figure S1). To explore the evolutionary relationships and classification of cucumber PLATZ proteins, a phylogenetic tree was constructed using the amino acid sequences of PLATZ proteins from cucumber, Arabidopsis, and watermelon with FastTree with the maximum likelihood (ML). As shown in Figure 1A, PLATZ proteins were clustered into five groups.

The intron number of cucumber *PLATZ* genes ranged from two to four. *PLATZ2* had two introns, while nine cucumber *PLATZ* genes (*PLATZ1*, *3*-*8*, *10*, and *12*) had three introns; *PLATZ9* and *PLATZ11* had four introns (Figure S2). Eight *PLATZ* genes (*PLATZ2*, *3*, *4*, *5*, *6*, *7*, *9*, and *10*) exhibited three exons, while four *PLATZ* genes (*PLATZ1*, *8*, *11*, and *12*) displayed four exons (Figure S2). To determine the motif compositions of the cucumber PLATZ proteins, a total of ten conserved motifs were identified using MEME (https://meme-suite.org/meme/tools/meme (accessed on 1 September 2023) (Figure S2). Among the cucumber PLATZ proteins, PLATZ9 had four conserved motifs, while six PLATZ proteins (PLATZ2, 3, 6, 8, 10, and 11) had six conserved motifs; PLATZ4 had seven conserved motifs; four PLATZ proteins (PLATZ1, 5, 7, and 12) had eight conserved motifs (Figure S2). Gene duplication events have consistently been accompanied by the expansion and formation of gene families. The Multiple Collinearity Scan toolkit (MCScanX) and BLASTP were employed to investigate the gene duplication events of cucumber *PLATZ* genes. As illustrated in Figure S3A, there was a single pair of segmental duplication genes (*PLATZ5* and *PLATZ7*) on chromosome 3, and no tandem duplication events were identified. To further analyze the phylogenetic mechanisms of the *PLATZ* genes between cucumber, watermelon and Arabidopsis, a comparison of syntenic maps was conducted. Between cucumber and Arabidopsis, there were 12 pairs of collinear *PLATZ* genes were identified (Figure S3B).

Cis-acting regulatory elements (CREs) are regulatory sequences located in gene promoter regions involved in the regulation of downstream gene expression and are responsible for binding with transcription factors. The promoter regions (3000 bp upstream of the translation initiation site) of cucumber PLATZ were used to investigate the CREs in the cucumber PLATZ promoters. The CREs in promoters of cucumber *PLATZ* genes were explored using PlantCARE (https://bioinformatics.psb.ugent.be/webtools/plantcare/html/ (accessed on 15 September 2023). We identified a total of 24 CREs in the promoters of cucumber PLATZ genes (Figure 1B). The CREs of cucumber *PLATZ* genes were found to be involved in a number of biological processes, including light response, hormone response (gibberellin, auxin, salicylic acid, and MeJA), stress response, low-temperature responsiveness, and circadian control (Figure 1B). Notably, these signals are pivotal for plant assimilate loading. The results of the CRE analysis indicated that these signal pathways could be implicated in the regulation of PLATZ expression.

### 2.2. The Expression of RFO Synthesis Genes in PLATZ-RNAi Plants

**Figure 2:**
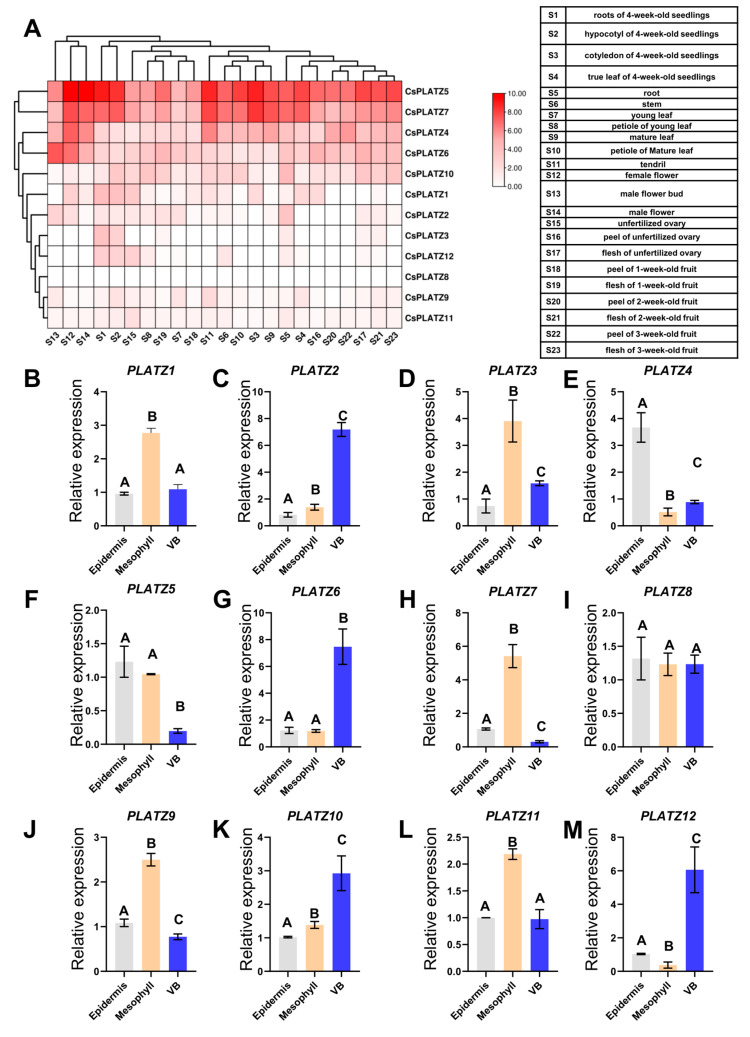
The expression of PLATZ members in cucumber. (**A**) The expression level of *PLATZ* genes in twenty-three tissue samples from cucumber. The heatmap was conducted based on the FPKM (Fragments Per Kilobase of exon model per Million mapped fragments) of public transcriptome data (PRJNA312872). (**B**–**M**) The expression level of cucumber *PLATZ* genes wasanalyzed using qRT-PCR in the mesophyll, epidermis and vascular bundle (VB). The tissues were collected from the seventh leaves from the top of 6-week-old cucumber plants and separated with forceps. Error bars denote standard deviations calculated from three biological replicates. Bars marked with different letters show statistically significantly difference (*p* < 0.01), as determined using Fisher’s least significant difference test after ANOVA. The relative gene expression levels were normalized using *Cs18S*.

The synthesis of RFO in the phloem was mainly observed in the leaves; thus, the expressions of cucumber *PLATZ* genes in cucumber tissues were further analyzed. A total of 23 previous transcriptome data points of cucumber tissues were used to analyze the expression patterns of cucumber *PLATZ* genes. The results demonstrated that *PLATZ* genes displayed no obvious sign of tissue specificity (Figure 2A, Table S2). To further investigate the expression of cucumber *PLATZ* genes in leaves, mesophyll tissue, epidermal tissue and vascular bundle tissue were isolated. Five *PLATZ* genes (*PLATZ 1*, *3*, *7*, *9*, and *11*) were strongly expressed in mesophyll tissue (Figure 2B–M). *PLATZ4* was highly expressed in epidermal tissue (Figure 2E). *PLATZ5* was prominently expressed in both mesophyll tissue and epidermal tissue (Figure 2F). *PLATZ2*, *PLATZ6*, and *PLATZ12* were highly expressed in vascular bundle tissue (Figure 2B–M).

**Figure 3:**
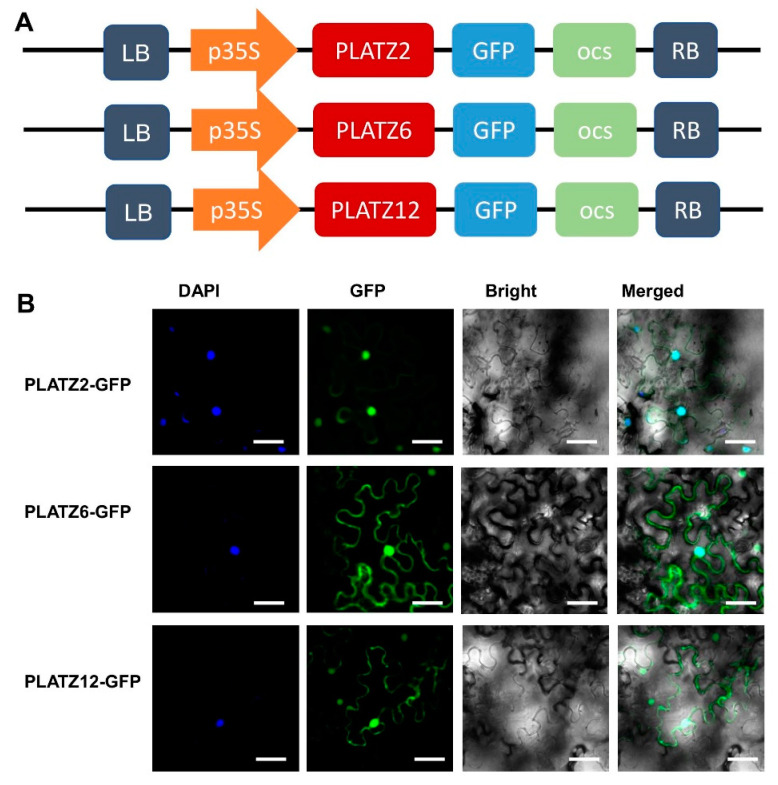
The subcellular localization analysis of cucumber PLATZ proteins. (**A**) Schematic diagram represents 35S:CsPLATZ2-GFP, 35S:CsPLATZ6-GFP, and 35S:CsPLATZ12-GFP. (**B**) Subcellular localization of three cucumber PLATZ proteins. DAPI signal show the nuclear. Bars = 100 μm.

To identify the cucumber *PLATZ* genes regulating RFO synthesis in leaf phloem, three *PLATZ* genes with a high expression in vascular bundle tissue were further characterized. To further understand the PLATZ2, PLATZ6, and PLATZ12 proteins characteristics of gene expression regulation, a subcellular localization assay was conducted. The fusion protein of PLATZ and green fluorescent protein (GFP) was expressed in tobacco leaves, and the GFP signal was observed to present the location of PLATZ in the cell. As shown in Figure 3B, GFP signals from the expressed fusion PLATZ2-GFP, PLATZ6-GFP, and PLATZ12-GFP were distributed in the cytomembrane and cell nucleus as confirmed with DAPI (DNA dye 4, 6-diamidino-2-phenylindole) staining. The results indicated that PLATZ2, PLATZ6, and PLATZ12 were partially expressed in the nucleus, and might regulate gene expression in the nucleus.

**Figure 4:**
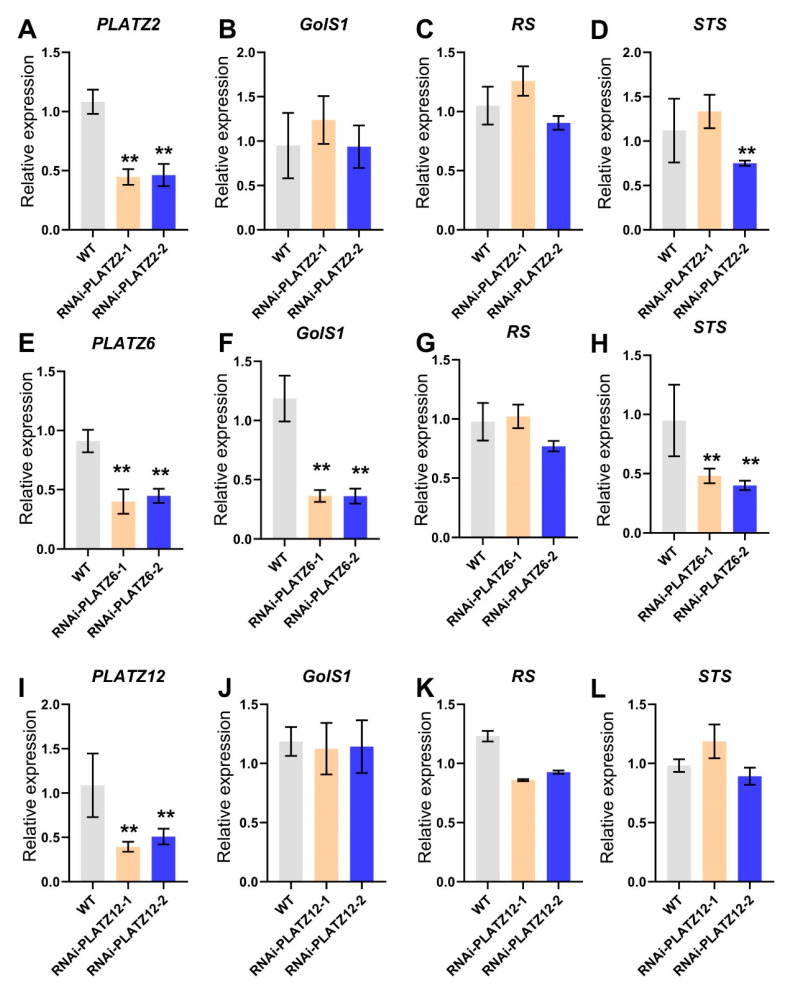
RFO synthesis genes regulated by cucumber PLATZ6. (**A**,**E**,**I**) The expression of *PLATZ2*, *PLATZ6*, and *PLATZ12* in the leaves of VIGS plants. (**B**–**D**,**F**–**H**,**J**–**L**) The expression of RFO synthesis genes in the vascular bundle of the leaves of VIGS plants and the WT. The tissues were collected from the seventh leaves from the tops of 6-week-old cucumber plants. Error bars denote the standard deviations calculated from three biological replicates. Bars marked with asterisks show statistically significantly differences (*p* < 0.01), as determined using Fisher’s least significant difference test after ANOVA. The relative gene expression levels were normalized using *Cs18S*.

A virus-induced gene silencing (VIGS) assay was conducted to explore the role of PLATZ6 in RFO synthesis. As indicated in Figure 4, the expressions of *PLATZ2*, *PLATZ6*, and *PLATZ12* were significantly decreased in the corresponding RNAi plants. To validate the VIGS specificity, the expressions of the homologous genes in their VIGS plants were analyzed. In cucumber, PLATZ2 was the homologous gene of PLATZ6, and PLATZ1 was the homolog of PLATZ12 (Figure 1A). As shown in Figure S4, the expression of PLATZ6 in RNAi-PLATZ2 plants, PLATZ2 in RNAi-PLATZ6 plants, and PLATZ1 in RNAi-PLATZ12 plants showed no significanty difference compared with the WT, respectively. The expression of *GolS1*, *RS*, and *STS* were analyzed in RNAi-PLATZ plants. In RNAi-PLATZ2 and RNAi-PLATZ12 plants, the expressions of *GolS1*, *RS*, and *STS* exhibited no significant differences compared with the WT (WT refers to plants that were infiltrated with the empty vector control; Figure 4B–D,J–L). In RNAi-PLATZ6 plants, the expressions of *GolS1* and *STS* but not *RS* were significantly down-regulated (Figure 4F–H). These findings suggest that PLATZ6 may act as a positive regulator of RFO synthesis.

## 3. Discussion

Previous studies have shown that PLATZ proteins play an important role in multiple plant development processes. The high level of *AtPLATZ3* expression in roots suggests that they may be involved in the regulation of root development, nutrient transport, or ion homeostasis. Previous studies have demonstrated that *AtPLATZ3* is highly expressed in leaves and plays a role in promoting leaf growth by accelerating cell proliferation [29,30]. *AtPLATZ7* is expressed preferentially in the root, and the size of the root meristem can be controlled by ROS signals [31]. In rice, the PLATZ transcription factor regulates the expression of genes related to grain development. gl6/sg6 knockout mutants of PLATZ family members in rice showed a phenotype of shortened grain length and decreased grain weight, while *GL6*/*SG6* overexpressing plants significantly increased the grain size and weight, as well as increased the plant height and fresh plant weight [31,32]. *ORESARA15* (ORE15), a PLATZ transcription factor, plays a pivotal role in regulating leaf size and aging by directly regulating the expressions of *GRF1* and *GRF4* in the GRF/GIF family [29]. The maize embryo milk silty mutant *fl3* caused severe endosperm defects. The specificity of FL3 in starch endosperm cells is further demonstrated by its interaction with transcription factor class C 1 (TFC1) and RNA polymerase III subunit 53 (RPC53), which inhibits the transcriptional activity of the RNA polymerase III transcription complex [28,33]. In this study, our results show that PLATZ6 in cucumber might be involved in RFO synthesis in leaf phloem, which affects the plant’s organ growth and development.

Proteins frequently interact with other proteins. To analyze the interacting proteins of PLATZ6, STRING (https://cn.string-db.org (accessed on 10 September 2023) was employed to predict the interacting proteins of cucumber PLATZ6. The results of the STRING analysis indicated that PLATZ6 could interact with a set of DOF (DNA binding with One Finger) proteins (Figure S5). In plants, Dof transcription factors are involved in regulating multiple physiological and biochemical processes, including plant tissue differentiation, seed germination, and substance metabolism. In addition, Dof transcription factors primarily regulate key genes and photosynthetic metabolites in the photosynthetic pathway of plant carbon metabolism. The overexpression of the maize *ZmDof1* gene can improve the activity of the C4pepc promoter in the maize protoplasm, and increase the activity of the cyppdk1 and non-photozygous *pepcZm2A* promoter [34]. In addition, overexpressed *ZmDof1* in rice has been demonstrated to activate the expression of the PEPC gene, malate dehydrogenase coding gene (NADP-MAD) and isocitrate dehydrogenase coding gene (ICDH) dependent on nicotinamide adenine dinucleotide phosphate (NADP), thus promoting carbon metabolism [35]. Similarly, rice OsDof25 is a homologous gene of maize ZmDof1, and the overexpression of this gene also shows similar applications [36]. In addition, several CREs related to light response were identified (Figure 1A). Thus, *PLATZ6* might be regulated by light, and interact with DOF proteins to regulate RFO loading in leaf phloem.

There have been several reports on the involvement of environmental factors in regulating the output of assimilate from leaves. In plants, ABA plays a pivotal role in the regulation of the plant stress response. Recently, the NF-YB1-SLRL2-bHLH144 module regulated the ABA-mediated rice grain quality, and this suggested that ABA plays a crucial role in promoting rice quality [37]. In cucumber, *GolS1* can respond to several abiotic stresses including cold stress, and the overexpression of *GolS1* can significantly improve cold tolerance in cucumber [14]. In addition to to its function as a source of energy for photosynthesis, recent findings have revealed that light signaling may play an important role in regulating assimilate loading. In Arabidopsis, HY5, a light-signaling switch, regulates leaf assimilate loading [38]. We identified multiple CREs in the promoters of PLATZ that can respond to hormones, circadian control, and environmental signals such as light and low temperature. The CRE analysis indicated that these environmental signals may be involved in the regulation of cucumber leaf assimilate loading, and PLATZ may play an important role in these processes.

## 4. Materials and Methods

### 4.1. Plant Growth Conditions and Materials

Seeds from the cucumber cultivar “Jinyan 4” obtained from Tianjin Kerun Agricultural Technology Co., Ltd. (Tianjin, China). We soaked the cucumber seeds in 55 °C water for 20 min and placed them on moist filter paper at 28 °C for 48 h. Cucumber plants were cultured in an artificial climate chamber (a temperature of 25 °C/18 °C day/night, 14 h of light and 10 h of darkness, and 70% relative humidity). Tobacco (*Nicotiana. benthamiana*) seeds were grown in a growth cabinet under controlled temperature (22 °C) and light (16 h day/8 h night). The cucumber and tobacco plants were grown in organic cultivation soil (HXL001). The nutrient solution used was the Japanese Yamazaki cucumber nutrient solution formula: Ca(NO_3_)_2_·4H_2_O 826 mg/L, KNO_3_ 607 mg/L, NH_4_H_2_PO_4_ 115 mg/L, MgSO_4_·7H_2_O 483 mg/L, FeSO_4_ 13.9 mg/L, EDTA-Na 218.6 mg/L, H_3_BO_3_ 2.86 mg/L, MnSO_4_ 2.13 mg/L, ZnSO_4_ 0.22 mg/L, CuSO_4_ 0.08 mg/L, and (NH4)_6_Mo_7_O_24_·4H_2_O 0.02 mg/L. Cucumbers were watered with nutrient solution at one-leaf stage, and 100 mL of nutrient solution was poured into each bowl every 4 d. Thirty-day-old seedlings were used for the gene expression analyses.

### 4.2. Subcellular Localization Assay

The subcellular localization assay of PLATZ2, PLATZ6, and PLATZ12 was conducted as described previously [39]. For overexpression of PLATZ-GFP in tobacco, the assay was conducted as previously described [40]. The CDSs of *PLATZ* genes were infused with GFP driven by the 35S promoter. The OE-PLATZs-GFP were transformed into *Agrobacterium tumefaciens* GV3101 competent cells and transiently expressed in the leaves of 4-week-old tobacco plants. After 48 h of culture, the leaves were sampled and immersed in a 4,6-diamidino-2-phenylindole (DAPI) staining solution containing 1 μg mL^−1^ DAPI for 10 min, and the GFP signal was observed and imaged using confocal microscopy (Germany, LSM 880 Zeiss).

### 4.3. PLATZ Family Members Identification in Cucumber

The Cucurbit Genomics Database was used to download the complete cucumber genome sequences from version 3 (http://cucurbitgenomics.org/organism/20 (accessed on 1 September 2023). The PLATZ sequences of watermelon and Arabidopsis were downloaded from the Cucurbit Genomics Database (http://cucurbitgenomics.org/organism/21 (accessed on 1 September 2023) and the Arabidopsis genome database (https://www.arabidopsis.org (accessed on 1 September 2023). We used the amino acid sequences of Arabidopsis PLATZ genes as queries for BLAST analysis in TBtools (e-value, 1 × 10^−10^). Then, PLATZ domain profiles (PF04640) were obtained from the Pfam database (http://pfam.xfam.org/ (accessed on 3 September 2023), and we identified the cucumber PLATZ genes of the Cucumber Genome Database (v3.0) using HMMER 3.0.

### 4.4. Phylogenetic Analysis

The PLATZ protein sequences of cucumber, Arabidopsis, and watermelon were used to perform a phylogenetic analysis. We used the Clustal W program (the default parameters) and neighbor-joining (NJ) tree to build the phylogenetic tree. Interactive Tree of Life (ITOL, https://itol.embl.de/itol.cgi (accessed on 1 September 2023) was employed to visualize and refine the phylogenetic tree.

### 4.5. Gene Domain and Structure Analysis

For the analysis of the intron and exon structures of cucumber *PLATZ* genes, the conserved motifs were characterized with MEME using the following default parameters (https://meme-suite.org/meme/tools/meme (accessed on 1 September 2023): we set the maximum number of motifs to 10, and the motifs optimum width was set to 6 to 100 residues. Batch-CD Search of TBtools with the default parameters was used to analyze the conserved domains. The exon and intron structures of the cucumber *PLATZ* genes were visualized using TBtools V2.

### 4.6. Cis-Regulatory Element Analysis of Cucumber PLATZ Promoters

In order to avoid missing the key elements of the promoter’s regulatory region of the gene, we selected the longer 3000 bp for promoter analysis. There are several reports suggesting that the UTR has an important role in regulating gene expression, and they include the UTR in their promoter analysis [41,42]. Therefore, we analyzed the CREs on the 5′-UTR as well. We extracted 3000 bp of promoter regions upstream of the translation initiation site using TBtools with the default parameters. PlantCARE was used for the analysis of the cis-acting elements (CREs) in the promoter of cucumber *PLATZ* genes (http://bioinformatics.psb.ugent.be/webtools/plantcare/html/ (accessed on 3 September 2023). The cis-regulatory element analysis was visualized with TBtools V2.

### 4.7. Collinearity Synteny and Chromosomal Location Analysis

The orthologous *PLATZ* genes from Arabidopsis, watermelon, and cucumber were used for synteny relationship analysis. MCScanX and the multiple synteny plot tool of TBtools V2 with the default parameters were used to construct the collinearity maps. The gff3 files of the cucumber genome and Gene Structure View tool of TBtools with the default parameters were used to analyze the chromosomal locations of *PLATZ* genes in cucumber.

### 4.8. Gene Expression Analysis

Bioinformatic analysis of publicly available RNA-seq datasets were conducted as follows: the FPKM values of cucumber *PLATZ* genes were extracted from the published RNA-seq data (PRJNA312872) [43]. We selected twenty-three tissue samples from cucumber for transcript level analysis. The heatmap tool of TBtools was used to analyze the expression levels of cucumber *PLATZ* genes.

Mesophyll, epidermis, and vascular bundle (VB) tissues were collected from the seventh leaves from the tops of 6-week-old cucumber plants and separated with forceps. RNA extraction from the epidermis, mesophyll, and VB tissues was followed by qRT-PCR. The RNAiso Plus kit (TaKaRa, Dalian, China) was used to extract the total RNA from different tissues. The PrimeScript ™ RT Reagent Kit with gDNA Eraser (TaKaRa, Dalian, China) was employed. We used 1 ug of total RNA for cDNA synthesis. A One-Step SYBR PrimeScript RT–PCR kit (TaKaRa, Dalian, China) was used for real-time PCR analysis. We used the CFX96 Touch™ Real-Time PCR Detection System (Bio-Rad, Berkeley, CA, USA) for real-time PCR analysis (qRT–PCR). The 2^−∆∆Ct^ method was used to analyze the relative expression level. Cs18S was used for the normalization of the relative expression levels. We conducted three biological replicates for each experiment. The error bars signify the standard deviation of three biological replicates in the figures.

### 4.9. Virus-Induced Gene Silencing Assay

A VIGS assay was conducted as described previously [44]. Here, ~300 bp specific cDNA fragments of PLATZ2, PLATZ6, and PLATZ12 were cloned to the pTRSV RNA2S vector. The construct was transformed into GV3101 and subsequently transiently co-transformed into the leaves of a tobacco plant. The extracts of tobacco-infected leaves were used as inoculum for cucumber plants after 7 dpai (day post-agro-infiltration). The primers used in this assay are listed in the Table S1.

## 5. Conclusions

**Figure 5:**
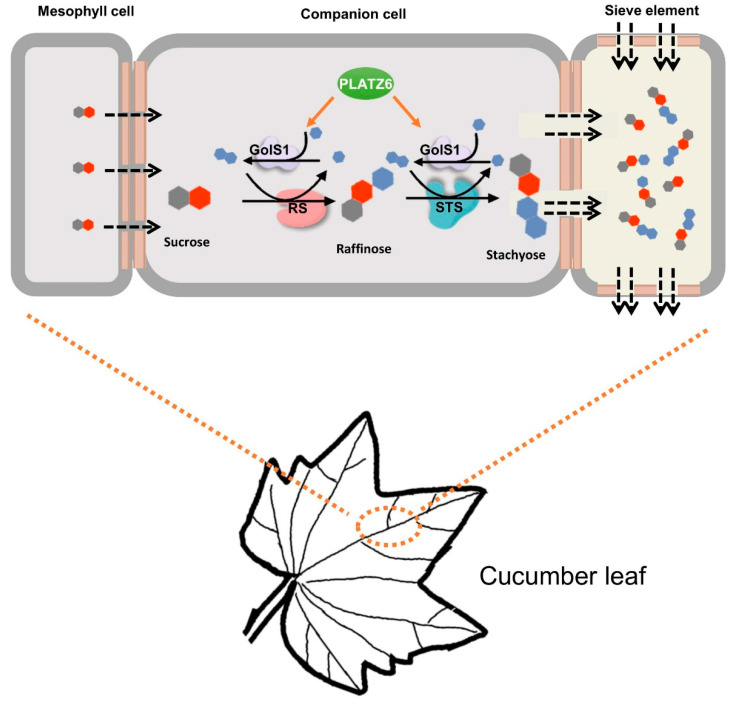
Proposed schematic diagram for the regulation of PLATZ6 with *GolS1* and *STS* gene. In cucumber leaves, *PLATZ6* was highly expressed in vascular tissues and regulated the expression of the *GolS1* and *STS* genes.

The *PLATZ* gene family represents a unique transcription factor family in plants and regulates various aspects of plant growth, development, and stress response. For the crucial role of PLATZ in plants, *PLATZ* genes have been identified in a number of plant species, including rice, Arabidopsis, tomato, wheat, and others. In this study, a total of 12 *PLATZ* genes were identified in the cucumber genome. The more comprehensive analysis of cucumber PLATZ identified that phloem that expressed PLATZ6 regulated the expression of *GolS1* and *STS*, two key enzymes of RFO synthesis (Figure 5). The results of this study will lay the foundation for the improvement of cucumber yield and stress resistance.

## Figures and Tables

**Table 1:** Physicochemical properties of PLATZ members in cucumber. ORF indicated open reading frame; MW indicated molecular weight; pI indicated theoretical isoelectric point; GAH indicated Grand Average of Hydropathicity.

Genes	Sequence ID	ORF (bp)	Size (aa)	MW (Da)	pI	Instability Index	Aliphatic Index	GAH	Predicted Location
CsPLATZ1	CsaV3_1G010140.1	774	257	28,996.08	8.18	55.35	69.42	−0.379	Nucleus
CsPLATZ2	CsaV3_1G014280.1	636	211	24,217.17	7.17	84.77	65.55	−0.684	Nucleus
CsPLATZ3	CsaV3_2G003600.1	732	243	27,674.93	8.99	59.97	76.13	−0.415	Nucleus
CsPLATZ4	CsaV3_2G036660.1	684	227	25,544.21	8.75	56.08	75.15	−0.529	Nucleus
CsPLATZ5	CsaV3_3G001590.1	657	218	24,770.08	9.38	62.13	65.69	−0.673	Nucleus
CsPLATZ6	CsaV3_3G036480.1	540	179	20,126.01	8.92	69.79	70.84	−0.384	Nucleus
CsPLATZ7	CsaV3_3G049080.1	663	220	24,909.59	9.45	57.59	71.27	−0.492	Nucleus
CsPLATZ8	CsaV3_5G004000.1	744	247	28,073.49	9.97	59.6	75.34	−0.413	Nucleus
CsPLATZ9	CsaV3_5G014490.1	423	140	15,597.68	9.06	64.14	58.43	−0.716	Nucleus
CsPLATZ10	CsaV3_5G027910.1	678	225	26,023.36	9.26	51.31	77.07	−0.534	Nucleus
CsPLATZ11	CsaV3_6G009240.1	750	249	27,449.47	8.82	56.71	75.58	−0.237	Nucleus
CsPLATZ12	CsaV3_6G052850.1	849	282	32,318.82	6.36	56.26	78.09	−0.437	Nucleus

## Data Availability

Data are contained within the article.

## Supplementary Materials

The following supporting information can be downloaded at: https://www.mdpi.com/article/10.3390/plants13192825/s1, Table S1. The primers used in this study. Table S2. Expression profiles of cucumber *PLATZ* genes based on RNA sequencing data. Figure S1. Phylogenetic analysis of PLATZ proteins in cucumber. (A) Location of the PLATZ members on the chromosomes of cucumber. The scale indicates the length of the chromosome. Figure S2. Gene structures and protein motifs of PLATZ transcription factor family in cucumber. (A) Ten protein motifs marked with colored box in cucumber PLATZ proteins. (B) Gene structure analyses of cucumber *PLATZ* genes. The green boxes show UTRs (untranslated regions); the yellow boxes show exons. Figure S3. Collinearity analysis of the PLATZ members. (A) Gene duplication events of PLATZ in cucumber. The outer circles indicated the chromosome distribution of cucumber *PLATZ* genes. The grey lines indicate all syntenic blocks of the cucumber genome. The purple lines of the outer ring represent the tandem duplication pairs of cucumber *PLATZ* genes, red lines show segmental gene duplication, and grey lines show all syntenic blocks in the cucumber genome. (B) Collinearity analysis of the PLATZ members between cucumber, Arabidopsis, and watermelon. Figure S4. The validation of VIGS specificity. Error bars denote the standard deviations calculated from three biological replicates. Bars marked with asterisks show statistically significantly differences, as determined using Fisher’s least significant difference test after ANOVA. The relative gene expression levels were normalized using Cs18S. Figure S5. Putative proteins interacting with cucumber PLATZ6. Putative interacting proteins predicted using STRING.
