# Supplementary figures and images for: The Role of PLATZ6 in Raffinose Family Oligosaccharides Loading of Leaves via PLATZ Family Characterization in Cucumber
Source: Plants (Basel). 2024 Oct 9;13(19):2825. doi: 10.3390/plants13192825 (PMC11478475; doi:10.3390/plants13192825)

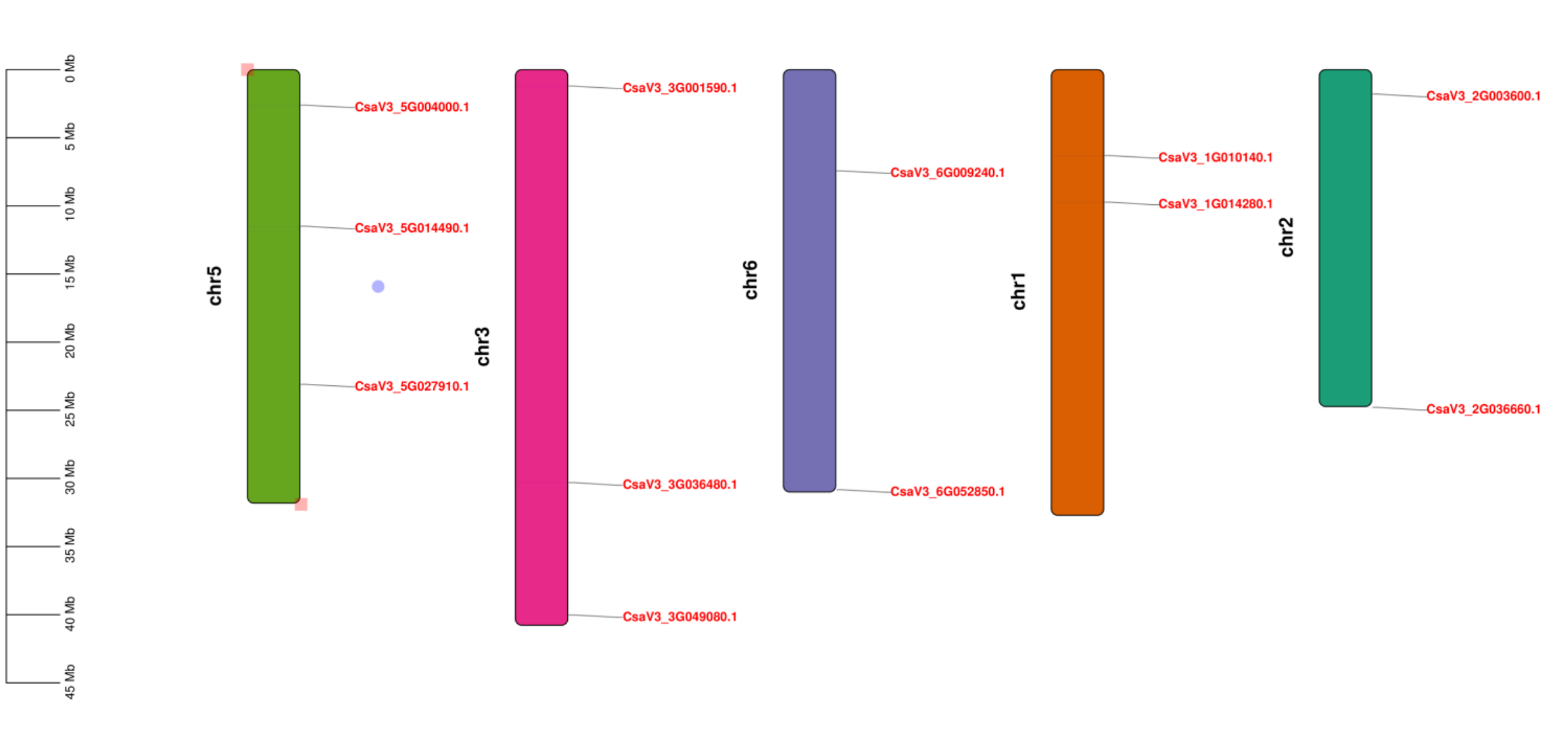

Supplement: Supplementary file 1 [file plants-13-02825-s001.zip › Figure S1_1.tif]

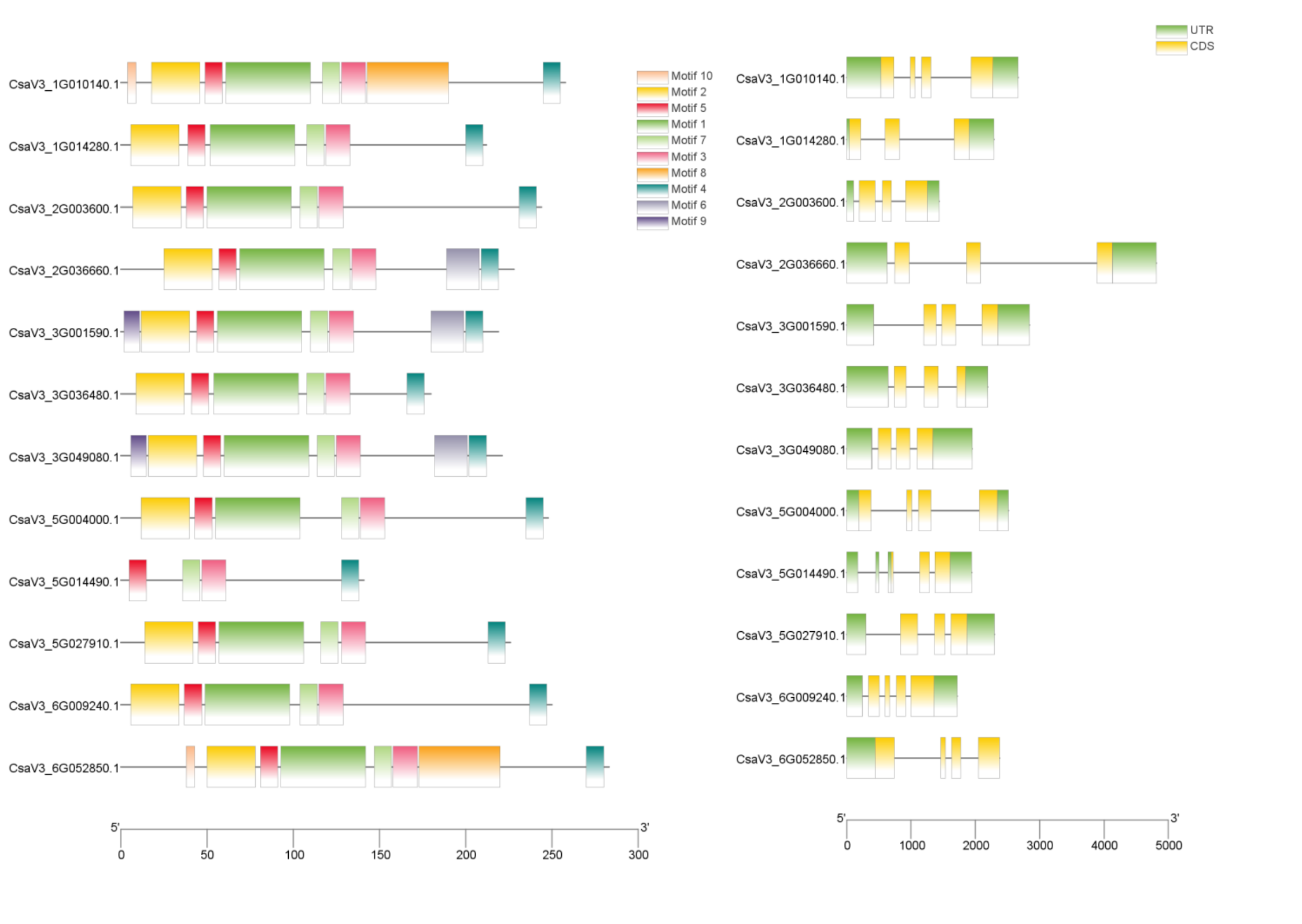

Supplement: Supplementary file 1 [file plants-13-02825-s001.zip › Figure S2_1.tif]

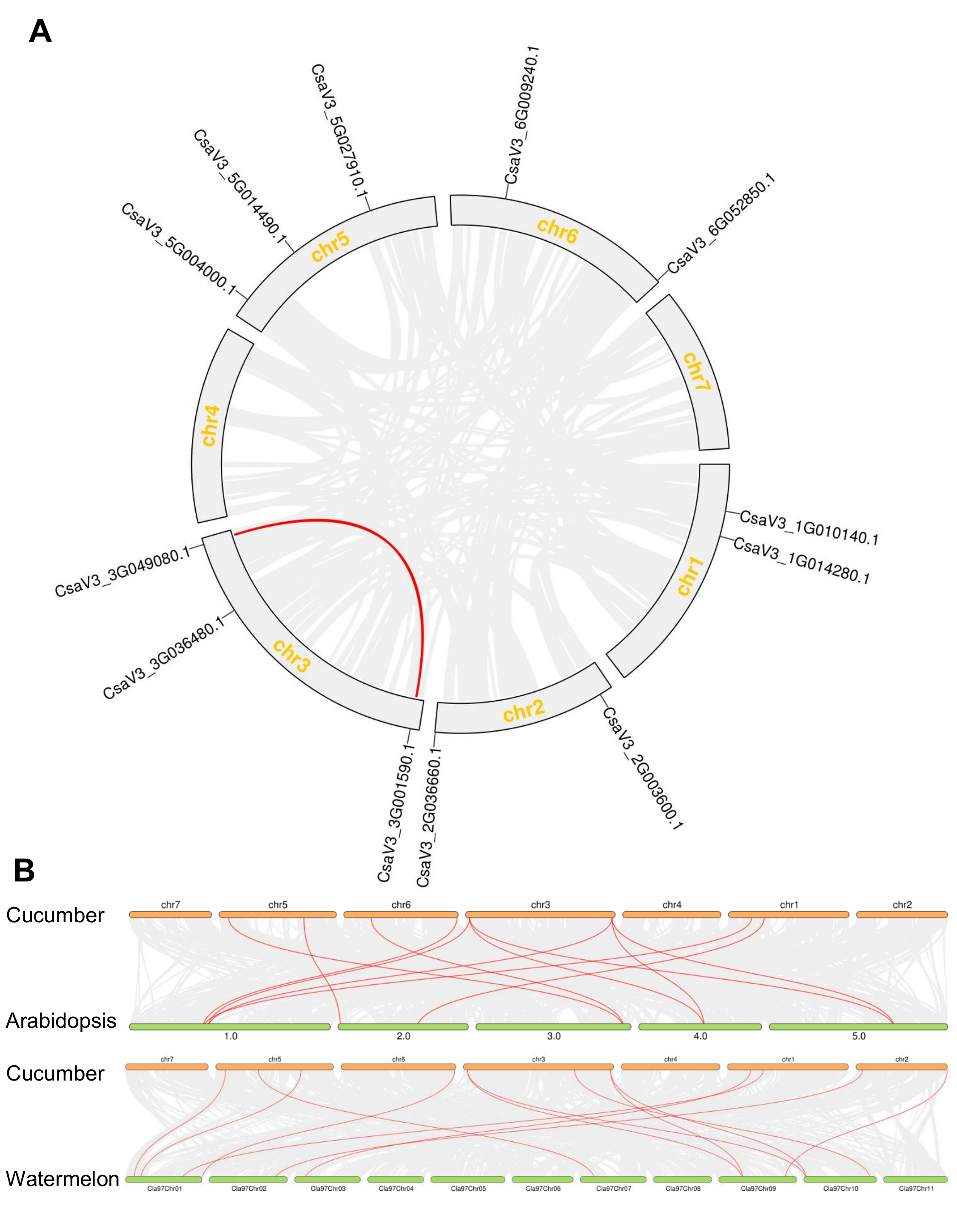

Supplement: Supplementary file 1 [file plants-13-02825-s001.zip › Figure S3_1.tif]

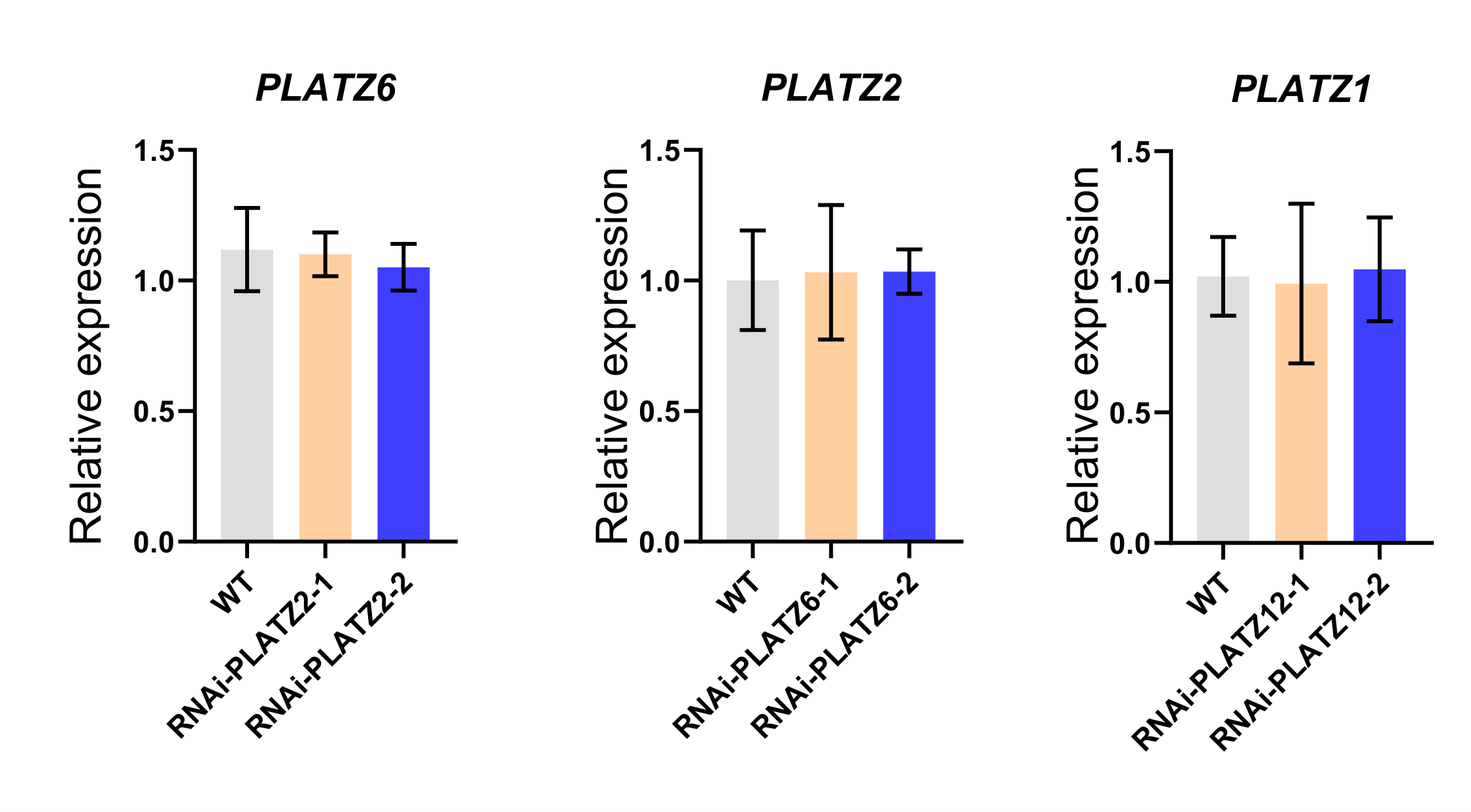

Supplement: Supplementary file 1 [file plants-13-02825-s001.zip › Figure S4_1.tif]

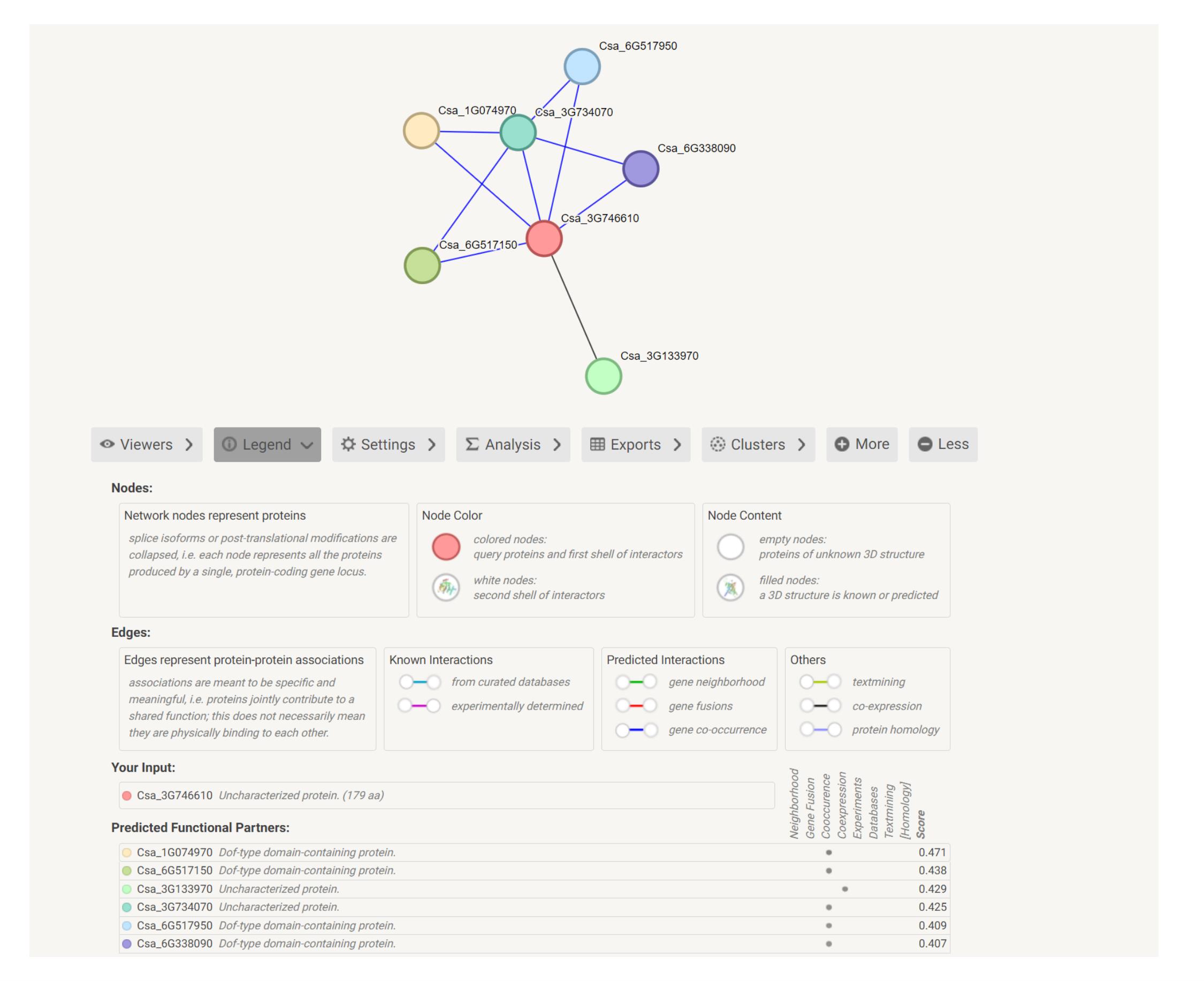

Supplement: Supplementary file 1 [file plants-13-02825-s001.zip › Figure S5. Putative proteins interacting with cucumber PLATZ6. Putative interacting proteins predicted by STRING.jpg]
